# IL-5 signaling in asthmatic derived fibroblasts exacerbates airway remodeling through ECM dysregulation and apoptosis resistance

**DOI:** 10.1186/s12931-025-03371-x

**Published:** 2025-11-04

**Authors:** Rola Abujabal, Tasneem M. Alanta, Reem Sami Alhamidi, Alaa Muayad Altaie, Lina Sahnoon, Bushra Mdkhana, Bassam Mahboub, Yves Laumonnier, Rifat Hamoudi, Khuloud Bajbouj, Qutayba Hamid

**Affiliations:** 1 Research Institute of Medical and Health Sciences, University of Sharjah, P.O. Box 27272, Sharjah, United Arab Emirates; 2 College of Medicine, University of Sharjah, Sharjah, United Arab Emirates; 3 Institute for Nutritional Medicine, University of Lübeck, Lübeck, Germany; 4 Center of Excellence for Precision Medicine, Research Institute of Medical and Health Sciences, University of Sharjah, P.O. Box 27272, Sharjah, United Arab Emirates; 5 Department of Pulmonary Medicine, Rashid Hospital, Dubai, United Arab Emirates; 6 Airway Research Center North, Member of the, German Center for Lung Research (DZL), Lübeck, Germany; 7 BIMAI-Lab, Biomedically Informed Artificial Intelligence Laboratory, University of Sharjah, P.O. Box 27272, Sharjah, United Arab Emirates; 8 ASPIRE Precision Medicine Research Institute Abu Dhabi, University of Sharjah, P.O. Box 27272, Sharjah, United Arab Emirates; 9 Division of Surgery and Interventional Science, University College London, London, WC1E 6BT UK; 10 Department of Biomedical Sciences, School of Veterinary Medicine, University of Pennsylvania, Philadelphia, PA USA; 11 Meakins-Christie Laboratories, McGill University, Montreal, Québec Canada

**Keywords:** Asthma, Airway Remodeling, Fibrosis, Interlukin-5 (IL-5)

## Abstract

**Background:**

Airway remodelling, a critical feature of severe asthma, involves fibroblast-driven extracellular matrix (ECM) dysregulation. While IL-5 is pivotal in eosinophilic inflammation, its direct role in fibroblast-mediated fibrosis remains undefined.

**Methods:**

Primary lung fibroblasts from asthmatic and healthy donors were stimulated with 0.5 ng/ml of IL-5 for several time points. ECM components, Matrix metalloproteinases (MMPs), Tissue inhibitor of metalloproteinases (TIMPs), and cytokines were analysed via quantitative real time-PCR (qRT-PCR), Western blot, ELISA, and flow cytometry. RNA sequencing and absolute gene set enrichment analysis (absGSEA) identified signaling pathways. Apoptosis was assessed using Annexin V/PI staining.

**Results:**

IL-5 shown to markedly increase the expression of ECM proteins, including collagen I and fibronectin, in asthmatic fibroblasts. It also upregulated MMP-2 and MMP-3 expression, alongside increased levels of TIMP-1 and TIMP-2. Moreover, IL-5 promoted the secretion of IL-6 and TGF-β. RNA-seq analysis identified 472 differentially expressed genes in asthmatic fibroblasts, highlighting activation of the MAPK pathway and suppression of apoptosis through NR4A1 upregulation. IL-5 further reduced fibroblast apoptosis and enhanced IL-5Rα expression, indicating potential autocrine signalling.

**Conclusion:**

IL-5 directly activates lung fibroblasts to drive airway remodelling in severe asthma through ECM deposition, MMP/TIMP imbalance, and pro-fibrotic cytokine secretion, positioning it as a dual mediator of inflammation and fibrosis with novel therapeutic potential.

**Graphical abstract:**

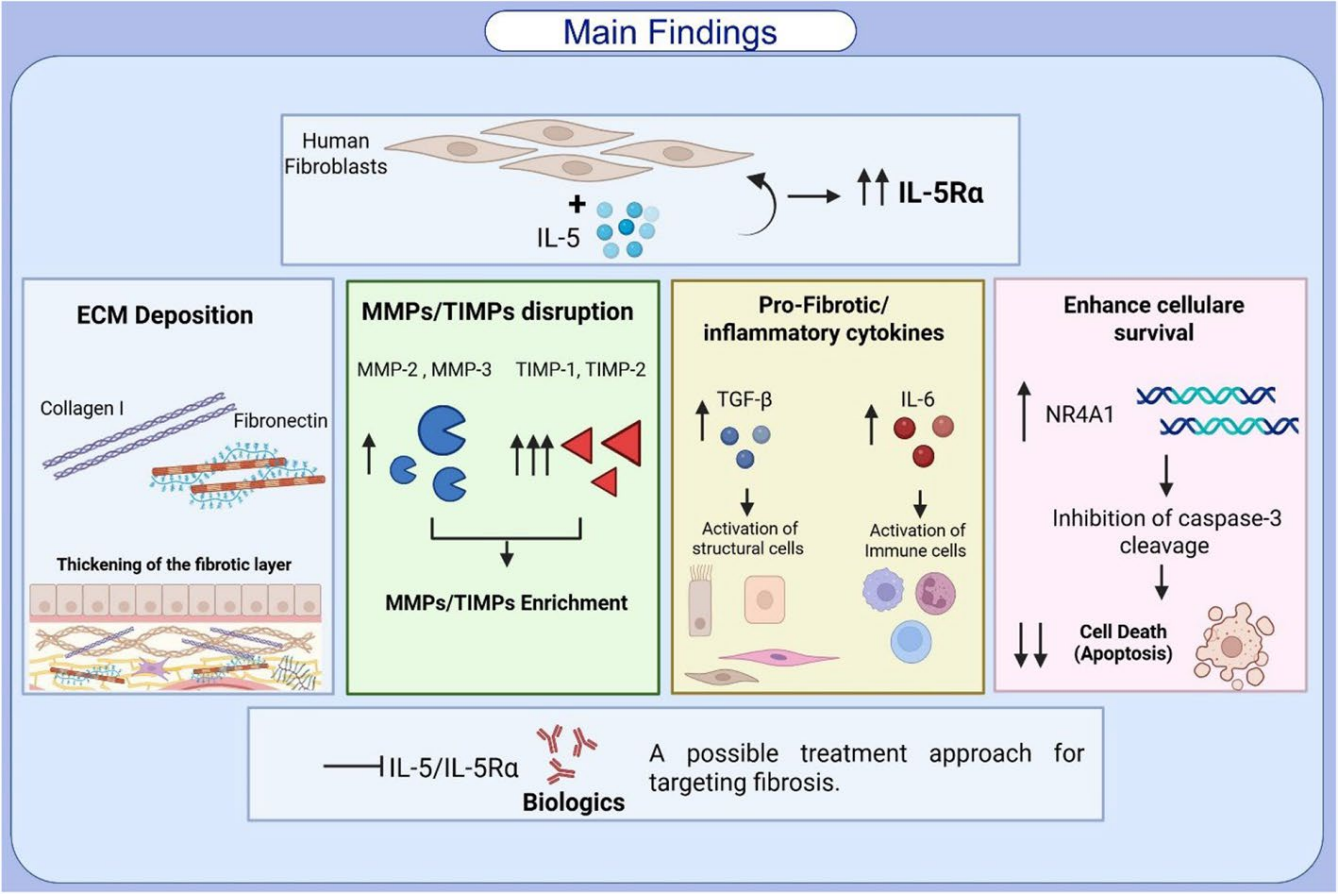

**Supplementary Information:**

The online version contains supplementary material available at 10.1186/s12931-025-03371-x.

## Introduction

Asthma is a chronic, multifactorial inflammatory lung disease characterized by variable airway obstruction due to an exaggerated type 2 immune response. This response, primarily mediated by T-helper 2 (Th2) cells, involves key cytokines such as interleukin (IL)−4, IL-5, and IL-13, which drive airway inflammation and hypersensitivity [1]. Chronic inflammation in asthma leads to aberrant tissue repair, resulting in structural alterations collectively termed airway remodelling [2]. Airway remodelling encompasses epithelial damage, subepithelial fibrosis, increased airway smooth muscle mass, goblet cell hyperplasia, and angiogenesis [3]. These changes contribute to irreversible or partially reversible airway narrowing and increased disease severity [4].

Among the pathological features of airway remodelling, subepithelial fibrosis is particularly critical due to its association with disease severity and airway stiffness. This process involves excessive deposition and impaired degradation of ECM components, including collagen I, III, and V, fibronectin, tenascin-C, and lumican, within the lamina reticularis beneath the basement membrane [4]. Dysregulation of ECM turnover results from an imbalance between matrix metalloproteinases (MMPs) and their natural inhibitors, tissue inhibitors of metalloproteinases (TIMPs) [2, 5]. Fibroblasts, the primary cellular constituents of the subepithelial layer, play a central role in fibrosis due to their biosynthetic, contractile, and pro-inflammatory properties [6]. Under the influence of transforming growth factor-beta (TGF-β), fibroblasts differentiate into myofibroblasts, which exhibit persistent activation in asthma, leading to excessive ECM deposition and fibrosis progression [7–9].

Interleukin-5 (IL-5), a key type 2 cytokine, is traditionally recognized for its role in eosinophil maturation, activation, and recruitment to sites of inflammation [9, 10]. However, emerging evidence suggests that IL-5 may also directly influence structural cells in the airway. Recent studies have identified IL-5 receptor alpha (IL-5Rα) expression on airway epithelial cells and fibroblasts, implicating IL-5 in processes beyond eosinophil-mediated inflammation [11, 12]. Despite these findings, the direct role of IL-5 in airway remodelling, particularly in fibroblast activation and fibrosis, remains poorly understood.

To evaluate this further, we investigated the direct effects of IL-5 on fibroblast activation and ECM remodelling, using primary airway fibroblasts cultured from endobronchial biopsies from patients with asthma. Specifically, we have assessed the impact of IL-5 on fibroblast proliferation, ECM deposition, MMP and TIMP expression, and cytokine secretion. Our findings provide novel insights into the pro-fibrotic role of IL-5 in asthma, highlighting its potential contribution to airway remodelling and suggesting new therapeutic targets and outcomes for intervention.

## Materials and Methods

### Fibroblasts cell culture

Human primary bronchial fibroblasts, obtained from Lonza derived from non-smoking subjects with asthma, and healthy individuals, were used in this study. The subject characteristics used in this study are summarized in (Supplementary Table 1). Cells were maintained in Gibco Dulbecco's Modified Eagle Medium: Nutrient Mixture F-12 (DMEM/F-12) (Cat: 10,565,018 Gibco-thermofisher) supplemented with 15% fetal bovine serum (FBS) (Sigma- Aldrich, Cat: F7524), 1% Penicillin/Streptomycin (Gibco®) and/or Fibroblast growth medium (FBM) from Lonza (Cat: CC-3132). Cells were cultivated in a 37 °C humidified incubator (Thermofisher) HERA cell 150i Carbon Dioxide Incubator) containing 5% CO_2_ and 21% oxygen. All experiments were performed using cells at passage 6 or earlier.

### Cell treatment

Asthmatic and healthy fibroblasts were stimulated with human recombinant Interleukin 5 (hrIL-5) (R&D, Cat: 205-IL-025) using 0.5 ng/ml for different time points based on each experiment need including 1 h, 6 h and 48 h concentration was chosen based on the previous work as in [11]. Cells were starved prior to stimulation using DMEM/F-12 (Cat: 10,565,018 Gibco-thermofisher supplemented with 0.01% FBS (Sigma Aldrich, Cat: F7524) and 1% Penicillin/Streptomycin for 24 h.

### Quantitative real time PCR (qRT-PCR)

Severe asthmatic and healthy fibroblasts were seeded at 0.5 × 10^5^ cells/mL in 6 well plates till ~ 70% confluency. Cells then were collected and washed with ice cold PBS for RNA extraction. RNA extraction was done using Trizol (Cat: T9424, Sigma-Aldrich) following the manufacturer protocol. Complementary DNA (cDNA) was synthesized from 1 µg of total RNA using High-capacity cDNA synthesis kit (Cat: 4,368,814, Thermofisher) according to the manufacturer’s protocol. RT-PCR was performed using 100 ng/µl of cDNA, amplification was carried out using 5 × Hot FirePol EvaGreen qRT-PCR SuperMix (Cat: 08–36-00001, Solis Biodyne), amplification was done using QuantStudio 5 Real-Time PCR System (Applied Biosystems). The primers used are summarized in Table 1. Expression levels of the tested genes were normalized to 18 s expression and fold change was calculated following as 2^−ΔΔct^.

**Table 1:** Showing the primer sequences that was used in the qRT-PCR analysis

Gene Name	Forward	Reverse
*COL1A1*	5'-GATTGACCCCAACCAAGGCTG-3'	5'-GCCGAACCAGACATGCCTC-3'
*COL3A1*	5'-GATCAGGCCAGTGGAAATG-3'	5'-GTGTGTTTCGTGCAACCATC-3'
*COL5A1*	5'-GTCGATCCTAACCAAGGATGC-3'	5'-GAACCAGGAGCCCGGGTTTTC-3'
*FN1*	5'-CTGGGAACACTTACCGAGTGGG-3'	5'-CCACCAGTCTCATGTGGTCTCC-3'
*IL6*	5'-AGACAGCCACTCACCTCTTCAG-3'	5'-TTCTGCCACTGCCTCTTTGCTG-3'
*TGFb*	5'-TACCTGAACCCGTGTTGCTCTC-3'	5'-GTTGCTGAGGTATCGCCAGGAA-3'
*TNF*	5'-CTCCTACCCGAACAAGGTCA-3'	5'-CGGTCACCCTTCTCCAACT-3'
*LUM*	5'-AACATACCAACTGTCAATGAAAACC-3'	5'-TGCCATCCAAACGCAAATGCTTG-3'
*TNC*	5'-TCCCAGTGTTCGGTGGATCT-3'	5'-TTGATGCGATGTGTGAAGACA-3'
*MMP7*	5'-GCTGGCTCATGCCTTTGC-3'	5'-TCCTCATCGAAGTGAGCATCTC-3'
*MMP2*	5′-CTCTCCTGACATTGACCTTGGCAC-3′	5′-AAAAAGCTTACTCGCTGGACATCAG-3′
*MMP3*	5’AAACTCCCGCGTCATAGAAA-3’	5’- TGAGTCAATCCCTGGAAAGT-3’
*MMP9*	5′-GGCATCCGGCACCTCTATGGTCC-3′	5′-GCCACTTGTCGGCGATAAGGAAGG-3′
*TIMP1*	5'-AGTCAACCAGACCACCTTATACCA-3'	5'-TTTCATAGCCTTGGAGGAGCTGGTC-3'
*TIMP2*	5'-TGCAATGCAGATGTAGTGATCAGGG-3'	5' -GCTTATGGGTCCTCGATGTCGAGA-3'
*IL5RA*	5′-CACACGCTGACTGTACTTGCAC-3′	5′-GGGCATTGAGAACGAACCTTA-3′
*NR4A1*	5’ ACTGCCAAACTGGACTACTC 3’	5’ AGAGCAGGTCGTAGAACTG 3’
*18 s*	5'-TGACTCAACACGGGAAACC-3'	5'-TCGCTCCACCAACTAAGAAC-3'

### Western blot

Cellular lysates of Human fibroblasts derived from healthy subjects, and severe asthmatics were used to evaluate the needed protein levels. Cells were lysed using ice-cold RIPA buffer (Cat: ab156034, Abcam) supplemented with protease cocktail inhibitor tablets (Sigma-Aldrich). Whole cell lysate protein concentrations were quantified using the BCA protein quantification kit following the manufacturer instruction Lysate aliquots containing 30 μg of protein was separated by 10% and 12% sodium dodecyl sulfate–polyacrylamide gel electrophoresis (SDS-PAGE) transferred onto a PVDF membranes (Cat: 1,620,177, Bio-Rad) post activation with 100% methanol. The membrane was blocked by 5% skimmed milk powder for 1 h at room temperature, washed with (1xTBST), and reacted with primary antibodies summarized in Table 2. Blots were probed with primary antibodies overnight at 4°C. The blot was then incubated with secondary (anti-mouse and anti-rabbit) antibodies (Cat: 7076S and 7074S Cell Signaling Technology) at 1:500 dilutions for 1 h at room temperature. Chemiluminescence was detected using the ECL kit (Cat: 170–5060 BioRad). Protein band quantification was carried out using the Bio-Rad Image Lab software (ChemiDoc™ Touch Gel and Western Blot Imaging System; Bio-Rad). Tubulin and GAPDH was used as a normalization control and to ensure equal loading.

**Table 2:** Summary of the primary antibodies used for protein detection

Protein	Description
IL-5Rα	Human IL-5 R alpha/CD125 Antibody, R&D, Cat: MAB253
Collagen I/COL1A1	Rabbit Monoclonal, CST, Cat No. 72026S
Fibronectin/FN1	Rabbit Monoclonal, CST, Cat No26836
Tenascin C	Rabbit Monoclonal, CST, Cat No. 33352S
MMP-2	Rabbit Monoclonal, CST, Cat No. 40994
MMP-3	Rabbit Monoclonal, CST, Cat No. 14351
MMP-7	Rabbit Monoclonal, CST, Cat No. 3801
MMP-9	Rabbit Monoclonal, CST, Cat No.13667
TIMP-1	Rabbit Monoclonal, CST, Cat No. 8946
TIMP-2	Rabbit Monoclonal, CST, Cat No. 5738
Caspase-3	Rabbit Monoclonal, CST, Cat No. 14220
GAPDH	Rabbit Monoclonal, CST, Cat No. 2118
Tubulin	Mouse Monoclonal, CST, Cat No, 2144

### Intracellular staining

Severe asthmatic and healthy lung derived fibroblast were seeded at 2 × 10^5^ in a 6 well plate, cells were left to reach 70% confluency, then starved overnight, then stimulated with 0.5ng/ml of hrIL-5 for 4 h. Brefeldin A (cat: 00–4506-51, Invitrogen), a blocker for the vesical formation to prevent protein secretion and trafficking was added 1 h before terminating the experiment, at a ratio of 1μL per 1mL of the culturing media. Cells were harvested and washed with ice-cold PBS. Cells were stained against allophycocyanin (APC) Anti Human IL-6 (Cat: 501,112, Biolegend), Alexa Fluor® 488 anti-human TNF-α Antibody (Cat: 502,915, Biolegend) and phycoerythrin (PE) anti-human LAP (TGF-β1) Antibody (Cat: 300,004, Biolegend). Antibodies were diluted in staining FACS buffer at 1:100 dilution, cells were incubated with the antibody mix for 30 min at 4 °C maintaining dark. Samples were processed using BD FACS Aria III (BD, USA) and analysed using FlowJo 10.0 software.

### ELISA

Severe asthmatic and healthy fibroblasts were seeded at 2 × 10^5^ in a 6 well plate, left to reach 70% confluency, then starved overnight, cells were treated or not with hrIL-5, for 4 h and 48 h, cell culture supernatant (media) was collected for ELISA purposes. ELISA kits were used be to detect the following secreted proteins: Human IL-6 ELISA Kit (Cat: ab178013; detection limit: 1.6 pg/mL), Human TGF-β1 ELISA Kit (Cat: ab100647; detection limit: 18 pg/mL), Human MMP-3 ELISA Kit (Cat: ab269371; detection limit: 4.4 pg/mL), and the Human MMP-3 Activity Assay Kit (Cat: ab118972).), following the manufacturer instructions.

### Immunofluorescence

Healthy and severe asthmatic cells were seeded at a density of 2 × 10^4^ cells/mL and cultured until reaching 70% confluency. The cells were then stimulated for 24 h with 0.5ng/ml of rh-IL-5, then cells were harvested and washed twice with PBS, followed by fixation in 4% paraformaldehyde for 20 min at room temperature (RT). After fixation, the cells were washed with PBS and permeabilized with 0.01% Triton X-100 in PBS for 10 min at RT. Blocking was performed using 3% BSA in PBS for 1 h at RT. Subsequently, the cells were incubated overnight at 4 °C with primary antibodies against IL-5Rα (Cat: MAB253, R&D Systems) Vimentin (Cat: 5741, Cell Signalling Technology), Rabbit mAb IgG XP® Isotype Control (Cat: 3900, CST), Mouse IgG isotype control (Cat: MAB002, R&D systems) at a 1:100 dilution. After washing with PBS, the cells were incubated with Alexa Fluor® 488-conjugated secondary antibody (Cat: 150,077, Abcam) for 1 h at RT. Excess secondary antibody was removed by washing with PBS. Genomic DNA was stained with 4′,6-diamidino-2-phenylindole (DAPI) (Cat: D1306, Invitrogen) according to the manufacturer's protocol. The slides were visualized using a Nikon confocal microscope (Nikon, Tokyo, Japan), and the mean fluorescence intensity was quantified using ImageJ software.

### RNA extraction

For Whole transcriptomic and RNAseq, Human lung fibroblasts, isolated from both asthmatic and healthy subjects, were seeded in 60 mm cell culture plates at a density of 2 × 10^5^ cells per plate. The cells were placed in a CO2-supplemented incubator (5% CO2) for 24 h to achieve 70% confluency for starvation and treatment with hr-IL5 for 6 h. Cells were trypsinized and harvested for RNA extraction. RNA extraction was carried on using Qiagen RNeasy extraction kit (Cat: 74,104, Qiagen Germany) and quantified using Nano-drop. RNA was subjected to Turbo DNase treatment using Turbo DNAase kit (Cat: AM1907, Invitrogen, USA) to eliminate all genomic DNA.

### Whole transcriptome

Whole transcriptome sequencing was performed using targeted RNA-Seq with Ion AmpliSeq™ whole transcriptome human gene expression kit (ThermoFisher Scientific, USA). Briefly, cDNA was synthesized using a SuperScript™ VILO™ cDNA Synthesis kit (ThermoFisher Scientific, USA) and amplified using Ion AmpliSeq gene expression core panel primers. The amplified products were subjected to enzymatic shearing to get amplicons of ~ 200bp, then ligated with the adapter and the unique barcodes. Next, the constructed library was purified using Agencourt AMPure XP Beads (Beckman Coulter, USA), quantified using an Ion Library TaqMan™ Quantitation Kit (Applied Biosystems, Waltham, USA), and further diluted to 100pM, and pooled equally with 16 individual samples. The diluted library was amplified and enriched using Ion Chef System (ThermoFisher Scientific) according to the manufacturer’s instructions. The prepared template libraries were sequenced using the Ion S5 XL Semiconductor sequencer with Ion 540 Chip.

### RNAseq data analysis

As previously described [13], transcriptome data analysis was conducted using the Ion Torrent Software Suite (version 5.4). Raw sequencing reads were first processed through preprocessing stages, including adapter trimming and the filtration of low-quality reads, using the built-in Ion Torrent tools. Reads were then aligned to the hg19 reference genome (GRCh37 assembly) using the Torrent Mapping Alignment Program (TMAP), which is explicitly tailor-made for Ion Torrent sequencing data. The alignment adapted a two-stage mapping strategy to maintain sensitivity and specificity. Firstly, the short reads were aligned via the Burrows-Wheeler Aligner (BWA-short), which efficiently handles short-read data. This was followed by using the Sequence Search and Alignment by Hashing Algorithm (SSAHA) to improve the alignments. Additionally, the BWA-long and Smith-Waterman algorithms were used to polish alignments by enabling super-maximal exact matching, particularly for complex regions. This joint approach ensured high confidence in mapped reads and reduced mapping errors inherent to Ion Torrent sequencing data. To ensure data reliability, a quality control check was performed on the expression of the selected genes including (*COL1A1, FN1, MMP3*). Differentially Expressed Genes (DEG) was performed with the DESeq2 package in R/Bioconductor using R (version 4.2), leveraging the raw RNA-seq data. Normalization to the raw read counts of the target genes were conducted using the Fragments Per Kilobase Million method. Genes with normalized counts below ten were excluded from subsequent analysis. DEGs were identified based on a significance threshold of *p* < 0.05 and were used for further downstream analysis in the gene set enrichment analysis (GSEA). Multiple testing correction was performed using the Benjamini-Hochberg (BH) procedure, and genes that passed the adjusted p-value threshold were identified. However, to provide a broader context for gene expression changes—and to avoid overlooking potentially biologically relevant genes due to the conservative nature of FDR correction in small sample sizes—we also considered genes with nominal *p*-values below 0.05.

### Gene set enrichment analysis

The significant DEG were obtained and further analysed to identify the activated and enriched cellular pathways in response to IL-5 stimulation in both normal and asthmatic derived lung fibroblasts using absolute GSEA (absGSEA with *p* < 0.05 and FDR < 0.25 as previously described [13, 14]. The absGSEA analysis was conducted on expression data using approximately 120,000 annotated cellular pathways from the Broad Institute's database (https://www.gsea-msigdb.org, accessed on 26 December 2024) along with custom-defined pathways from the gene set collections used (C1 to C8). In this study, Gene Ontology (C5) and immunologic gene sets (C7) gene set collections were the primary focus to obtain significantly enriched pathways. C5 and C7 represent the gene ontology (which includes biological processes, molecular functions and cellular processes) and the immunological pathways, respectively.

### Functional annotation and enrichment analysis

Functional clustering, annotation and pathway analysis was conducted using Metascape [15] (http://metascape.org, accessed on 26. December 2024). The frequently occurring genes generated from the GSEA analysis were used in Metascape to further validate the pathways identified in C5 and C7 results.

### Assessement of survival

Human lung fibroblasts, isolated from both asthmatic and healthy subjects, were seeded in 60 mm culture dishes at a density of 3 × 10^5^ cells per plate. The cells were placed in a CO_2_-supplemented incubator (5% CO_2_) for 24 h to achieve 70% confluency for starvation and treatment with hr-IL5 for 48h. All plates were then placed on ice, and the media were collected in labelled 15 mL tubes and kept on ice. Plates were washed twice with ice-cold PBS, followed by the addition of 700 µL of 1 × trypsin to each plate. Cells were centrifuged at 1000 RPM for 8 min at 4 °C and washed twice with PBS to remove any remaining media.

For apoptosis detection using the Annexin V-FITC Apoptosis Detection Kit (Abcam – ab14085), a master mix was prepared based on the number of samples following the manufacturer ‘s instructions. Briefly, a staining mixture was prepared in a 1.5 mL tube by combining Annexin V, PI, and binding buffer based on the number of samples. Each collected cell sample received 200 µL of the master mix, followed by gentle vortexing and incubation in the dark for 20 min at room temperature. The stained cells were then analyzed using a BD FACS III flow cytometer (Beckton Dickinson, USA), and the data were processed with FlowJo software.

### Statistical analysis

All data are presented as Mean ± Standard Deviation (SD) of 2–3 independent experiments. Data analyses were performed using paired two-tailed Student t- test to compare the control and treated fibroblasts using GraphPad Prism 8 software (GraphPad, San Diego, CA, USA) *p* < 0.05 was considered statistically significant. Microsoft office excel was utilized to obtain the log2FC and *p*-values from the conducted unpaired t-test on the RNA seq raw data to further validate the output from GSEA and gene frequency.

## Results

### Stimulation of asthmatic derived lung fibroblasts induce the secretion of ECM proteins

**Fig. 1:**
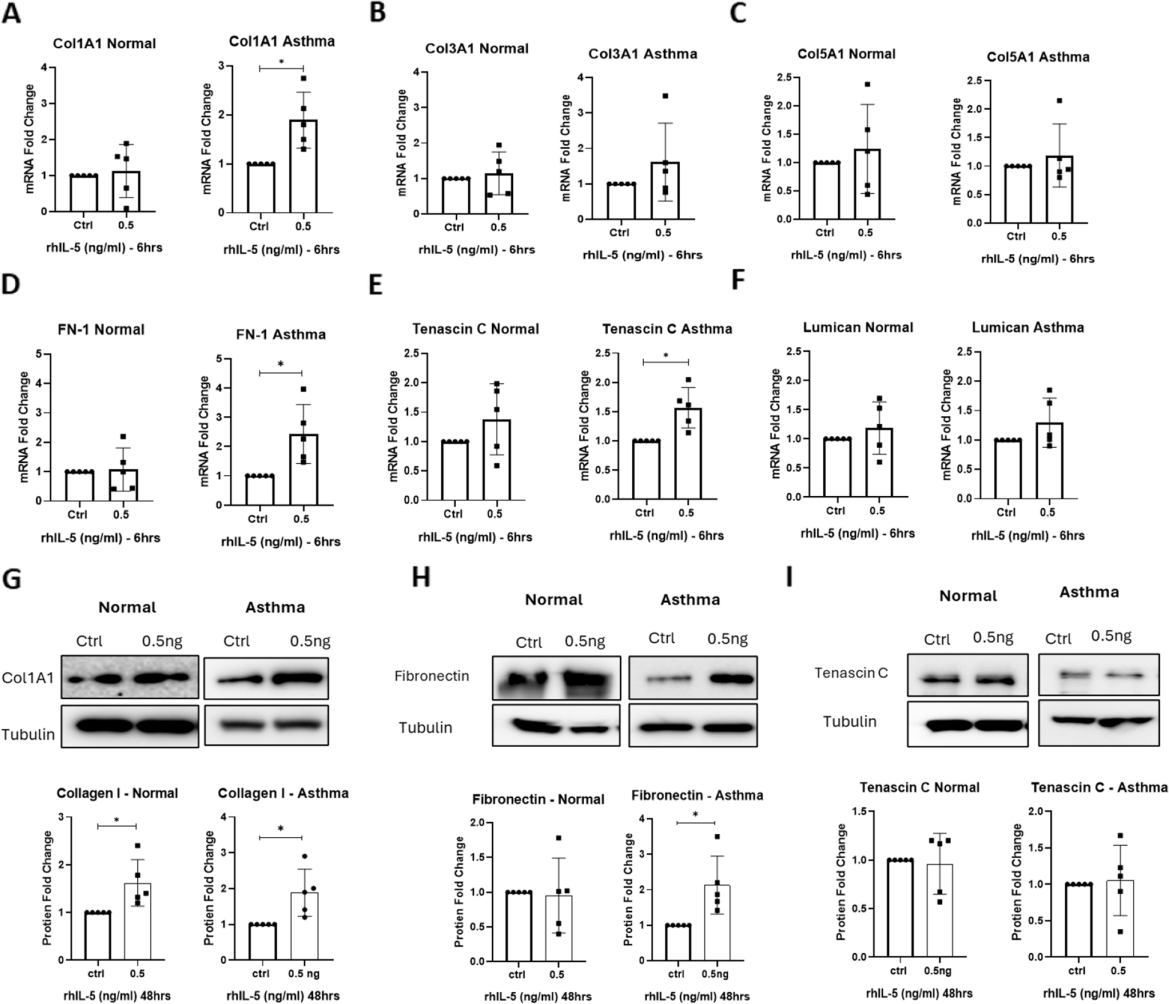
Evaluating the impact of fibroblasts stimulation with IL-5 on the levels of ECM genes and proteins. **A-F** mRNA levels of ECM genes in normal and asthmatic derived lung fibroblasts post stimulation with IL-5. *18s* was used as a housekeeping gene for ΔCt normalization, and fold changes upon IL-5 treatment compared to control was calculated as 2.^−ΔΔct^. Data shown are the mean fold changes ± SD of five separate experiment. Significance was assessed using two-tailed paired- student’sTest, *p < 0.05. (**A**) *COL1A1*, (**B**) *COL3A1*, (**C**) *COL5A1*, (**D**) *FN1*, (**E**) *Tenascin C*, and (**F**) *Lumican*. **G-I** Western immunoblots and densitometric analysis of expression in normal and asthmatic derived lung fibroblasts. **G** Collagen I, (**H**) fibronectin, (**I**) Tenascin C. Data shown are mean ± SD after normalization to the untreated control and are representative of five different experiments; Tubulin was used as loading control, significance was assessed using two-tailed paired t-Test, *p < 0.05

The gene expression profile was established using qRT-PCR measurements of mRNA from healthy control and asthmatic derived lung fibroblasts pre and post stimulation with IL-5 for 6 h. Stimulation of normal derived fibroblasts with IL-5 did not induce an upregulation of the expression of any of the ECM protein genes (Fig. 1A-F). However, stimulation of asthmatic lung fibroblasts with IL-5 significantly induced the expression of *Col1A1* (1.8952 fold, *p* = 0.0247*,* Fig. 1A), *FN-1* (2.42 fold, *p* = *0.0338*, Fig. 1D), and *TNC* (1.56 fold, *p* = 0.0218, Fig. 1E). These finding were further validated at the protein level using immunoblotting analysis, (Fig. 1G-I) showed the ECM components post stimulation with IL-5 for 48 h. Collagen I was evident at a higher significant level upon IL-5 stimulation in both normal (1.6 fold, *p* = 0.0466) and asthmatic (twofold, *p* = 0.0402) fibroblasts when compared to their unstimulated controls (Fig. 1G). Similarly, Fibronectin levels were also elevated in asthmatic (2.2 fold, *p* = 0.0363) fibroblasts as shown in Fig. 1H. Although Tenascin C showed a significant increase at the mRNA level, this was not corroborated at the protein level (Fig. 1I). These findings demonstrate that IL-5 may participate in ECM deposition by directly promoting the production of ECM proteins in asthmatic fibroblasts, a hallmark of subepithelial fibrosis.

### IL-5 increases the expression of MMP-3 and activity in asthmatic derived lung fibroblasts

**Fig. 2:**
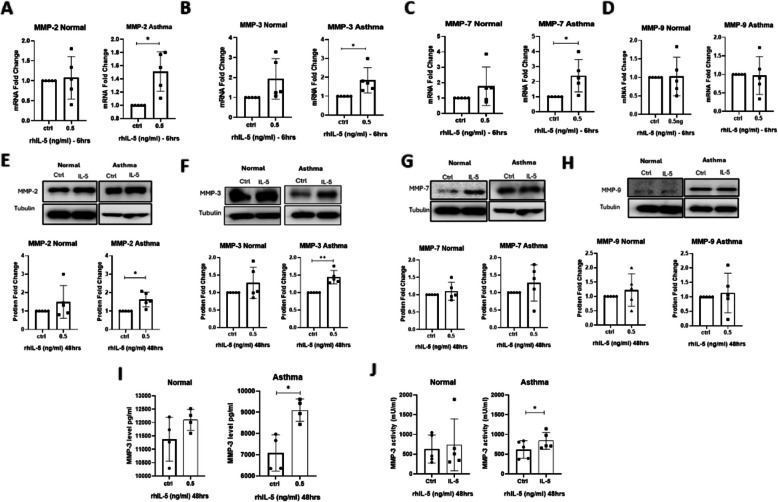
Evaluating the stimulatory effect of IL-5 on the levels of MMPs. **A-D** Showing the mRNA levels of the four MMPs in normal and asthmatic derived lung fibroblasts post stimulation with IL-5. Data shown is representative for five different experiments; *18s* was used as a housekeeping gene for ΔCt normalization, and fold changes upon IL-5 treatment compared to control was calculated as 2.^−ΔΔct^, Significance was assessed using two tailed paired student’s t-Test, *p < 0.05. **A** *MMP2*, (**B**) *MMP-3*, (**C**) *MMP-7*, and (**D**) *MMP9*. **E–H** Western immunoblots and densitometric analysis of expression in normal and asthmatic derived lung fibroblasts post 48 h of IL-5 stimulation. Data shown are mean ± SD after normalization to the untreated control and are representative of five different experiments; Tubulin was used as loading control, significance was assessed using two tailed paired student’s t-Test, *p < 0.05. **E** MMP-2, (**F**) MMP-3, (**G**) MMP-7, and (**H**) MMP-9. **I** Showing the secreted levels of MMP-3 from normal and asthmatic derived fibroblasts supernatant measured by ELISA. **J** Showing the activity levels of MMP-3 in both normal and asthma derived fibroblasts respectively measured by ELISA in a mU/ml. Data shown is the mean ± SD of at least four separate experiments; two tailed pared student’s t-Test statistical analysis was done *p < 0.05

MMPs and their tissue inhibitors TIMPs regulate ECM turnover. Dysregulation of this balance is a hallmark of fibrosis. To evaluate IL-5’s impact on MMP/TIMP expression, we performed qRT-PCR and Western blot analysis. In asthmatic fibroblasts, IL-5 stimulation significantly increased *MMP2* (1.63fold, *p* = 0.019), *MMP3* (1.82-fold, *p* = 0.0498), and *MMP7* (2.3-fold, *p* = 0.0448) but not *MMP-9* mRNA levels (Fig. 2A–D). Western blot analysis validated significant elevation of MMP-2 (1.44-fold, *p* = 0.0252) and MMP-3 (1.43-fold, *p* = 0.0068) protein expression in asthmatic fibroblasts with IL-5 stimulation (Fig. 2E–F), while MMP-7 and MMP-9 remained unaffected (Fig. 2G, H). Furthermore, ELISA measures revealed increased MMP-3 secretion in asthmatic (*p* = 0.0071) upon IL-5 stimulation (Fig. 2I), though enzymatic activity assays showed only a significant increase in asthmatic cells (*p* = 0.0155) (Fig. 2J).

**Fig. 3:**
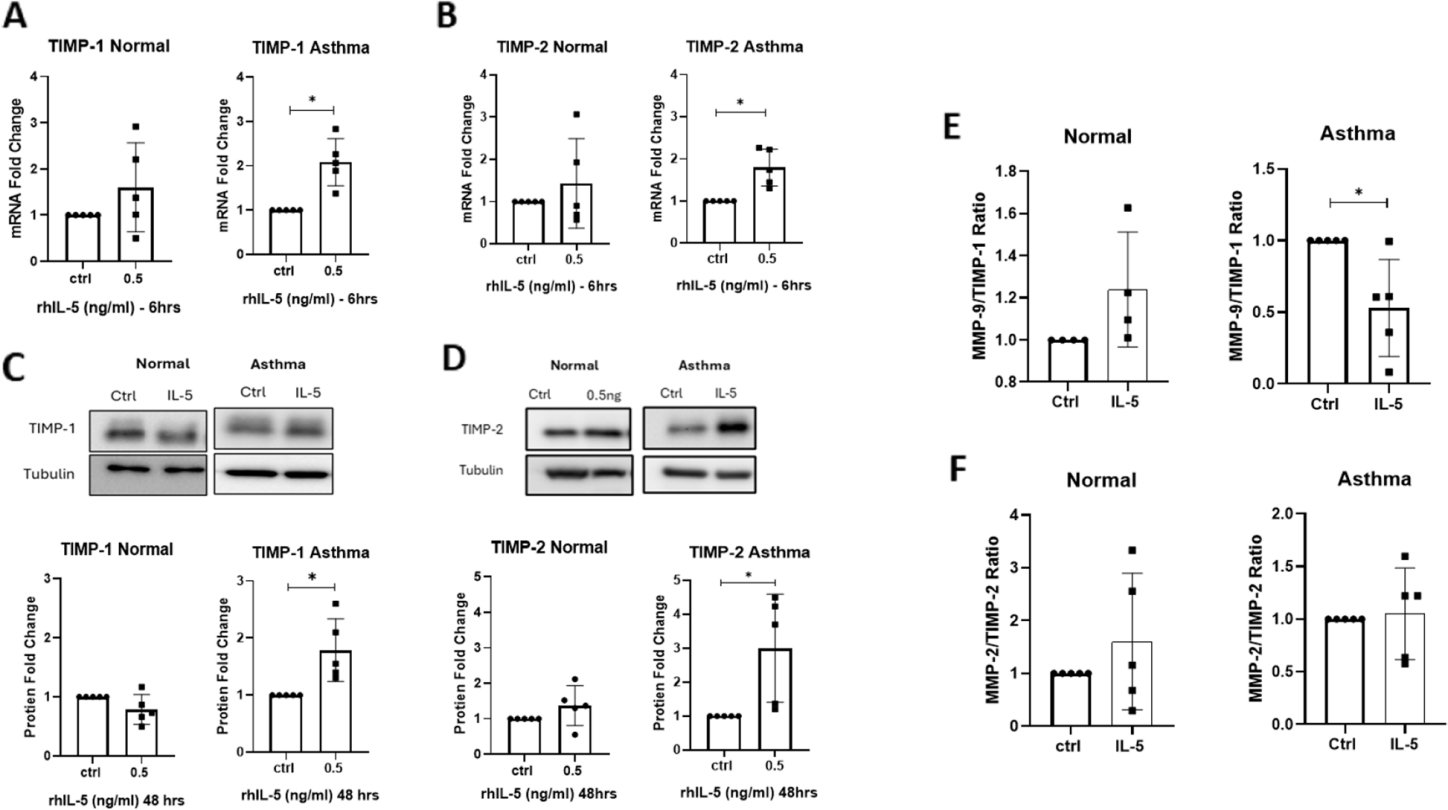
Lung fibroblasts stimulated with IL-5 have a higher level of TIMP-1 and TIMP-2. **A-B** showing the mRNA levels of both TIMP-1 and TIMP-2 in both Normal and asthma derived lung fibroblasts. *18s* was used as a housekeeping gene for ΔCt normalization, and fold changes upon IL-5 treatment compared to control was calculated as 2^−ΔΔct^. Data shown are the mean ± SD of five separate experiments; two-tailed paired student’s t-Test statistical analysis was performed *P < 0.05. **A** *TIMP1*, and (**B**) *TIMP2.* **C-D** Western immunoblots and densitometric analysis of expression in normal and asthmatic derived lung fibroblasts post 48 h of IL-5 stimulation. Data shown are the mean ± SD after normalization to the untreated control and are representative of five different experiments; Tubulin was used as loading control, significance was assessed using two-tailed paired t-Test, *p < 0.05. **C** TIMP-1, and (**D**) TIMP-2. **E** MMP-9/TIMP-1 ratios in normal and asthmatic fibroblasts in the presence of IL-5, significance was assessed using two tailed paired student’s t-Test, *p < 0.05. **F** MMP-2/TIMP-2 ratio in normal and asthmatic fibroblasts in the presence of IL-5, significance was assessed using two tailed paired student'st-Test

Concurrently, IL-5 upregulated *TIMP1* (2.1-fold, *p* = 0.0103) and *TIMP2* (1.8-fold, *p* = 0.0156) mRNA in asthmatic fibroblasts (Fig. 3A–B). Consistent with the mRNA data, both TIMP1 (1.8-fold, *p* = 0.0322) and TIMP-2 (3.01-fold, *p* = 0.0485) protein levels rising significantly in asthmatic fibroblasts when compared to their unstimulated controls (Fig. 3C-D). In consequence, we measured the MMP/TIMP ratio, we observed a marked shift in the MMP-9/TIMP-1 ratio toward TIMP-1 dominance (0.53-fold, *p* = 0.0359) in asthmatic fibroblasts (Fig. 3E), suggest a possible impaired ECM degradation by asthmatic fibroblasts. These results highlight IL-5’s role in dysregulating the MMP/TIMP equilibrium in asthmatic fibroblasts which might contribute further to fibrosis.

### IL-5 upregulated the expression of inflammatory and fibrotic cytokines in asthmatic lung derived fibroblasts

**Fig. 4:**
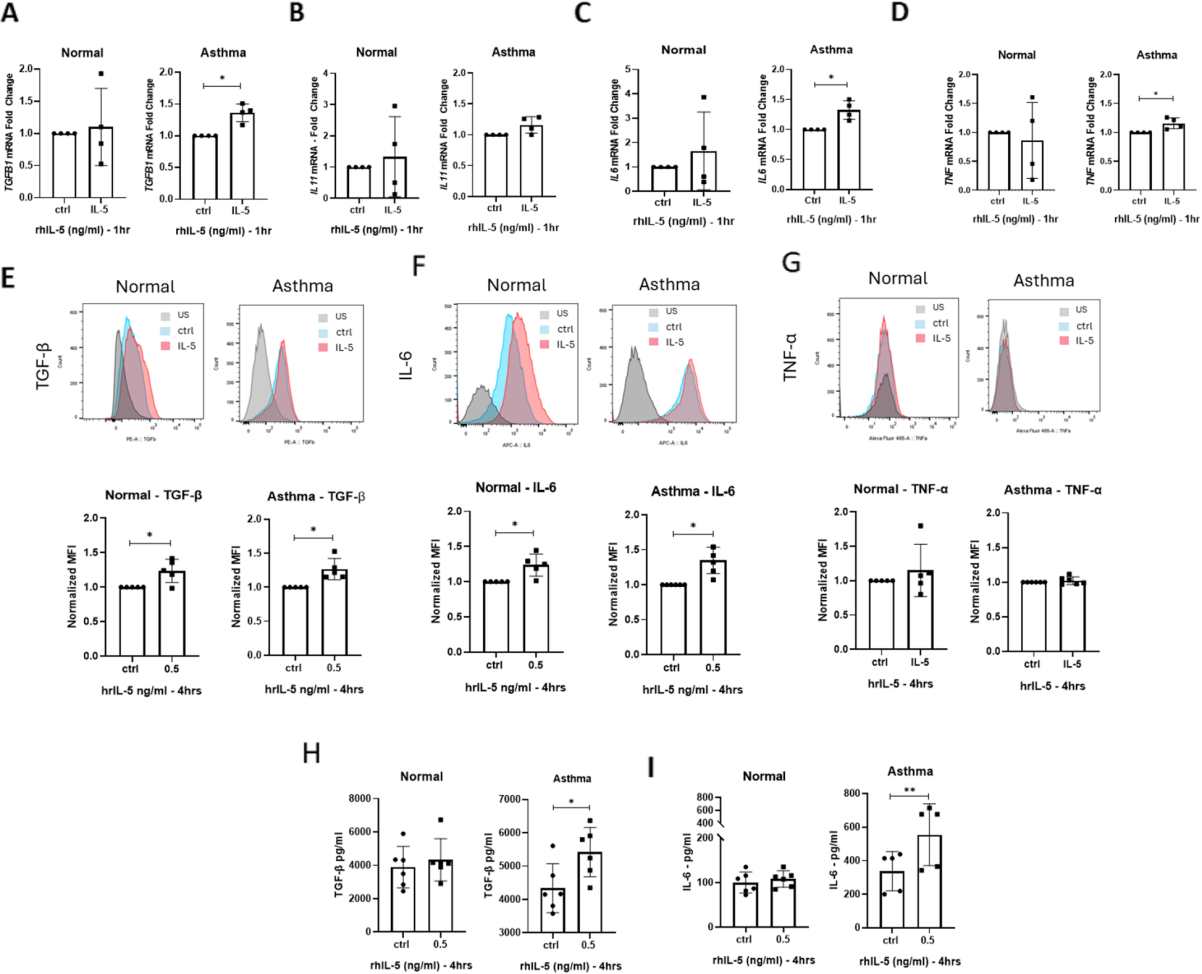
IL-5 promotes the expression of profibrotic and proinflammatory cytokine in asthmatic fibroblasts. **A-D** Expression of cytokines mRNA upon IL-5 stimulation (**A**) mRNA levels of *TGF-β,* (**B**) mRNA levels of *IL11*, (**C**) mRNA levels of *IL6*, and (**D**) mRNA levels of *TNF-α* in normal and asthma derived lung fibroblasts post 1 h of IL-5 stimulation. For all, *18s* was used as a housekeeping gene for ΔCt normalization, and fold changes upon IL-5 treatment compared to control was calculated as 2.^−ΔΔct^. Data shown are the mean of the fold change between IL-5 stimulated compared to control ± SD of three separate experiment; paired two-tailed student’s t-Test statistical analysis was done *p < 0.05*.* **E–F** Flow cytometric analysis of intracellular expression of cytokines. Histogram show expression in unstimulated (blue histogram) or IL-5-stimulated (red histogram) cells. Graphs show the mean expression in IL-5 stimulated cells normalized to control ± SD of five separate experiments for (**E**) IL-6, (**F**) TGF-β, and (**G**) TNF-α. Significance was assessed using two tailed paired student’s t-Test, *p < 0.05. **H** Showing the secreted levels of TGF-β from normal and asthmatic derived fibroblasts supernatant measured by ELISA, (**I**) Showing the secreted levels of IL-6 from normal and asthmatic derived fibroblasts supernatant measured by ELISA. Data shown are the mean of the fold change between IL-5 stimulated compared to control ± SD of six separate experiment; paired two-tailed student’s t-Test statistical analysis was done *p < 0.05, **p < 0.01

Fibroblasts contribute to airway inflammation by secreting cytokines and growth factors. To investigate IL-5’s effect on cytokine expression, we assessed mRNA and protein levels of key cytokines. At 1-h post-stimulation, *TGFB1* (1.4-fold, *p* = 0.014mRNA levels were significantly higher in asthmatic fibroblasts upon IL-5 exposure (Fig. 4A). Similarly, *IL6* (1.32-fold, *p* = 0.025) and *TNF* (1.16-fold, *p* = 0.046) were significantly upregulated in asthmatic fibroblasts upon IL-5 exposure (Fig. 4C–D). Flow cytometry analysis was conducted, the gating strategy was performed as in Supplementary Fig. 1, confirming the increase in the intracellular levels of TGF-β (*p* = 0.0187) and IL-6 (*p* = 0.0063) in asthmatic fibroblasts (Fig. 4E–F). Notably, healthy fibroblasts exhibited paradoxical increases in TGF-β (*p* = 0.03) and IL-6 (*p* = 0.028) and protein levels despite unchanged mRNA levels (Fig. 4E–F). Finally, secreted TGF-β (*p* = 0.012) and IL-6 (*p* = 0.0021) were significantly elevated in IL-5 asthmatic supernatants compared to the unstimulated control (Fig. 4H–I), however, normal cells didn’t show a significant secreted levels of either TGF- β or IL-6, this could be explained due to regulatory mechanisms exerted by the normal cells to resist the effect of IL-5. Altogether, our data indicates that IL-5 plays a role in amplifying inflammatory and fibrotic signalling cascades in asthmatic fibroblasts.

### IL-5 enhances the expression of IL-5Ra on lung derived fibroblasts

**Fig. 5:**
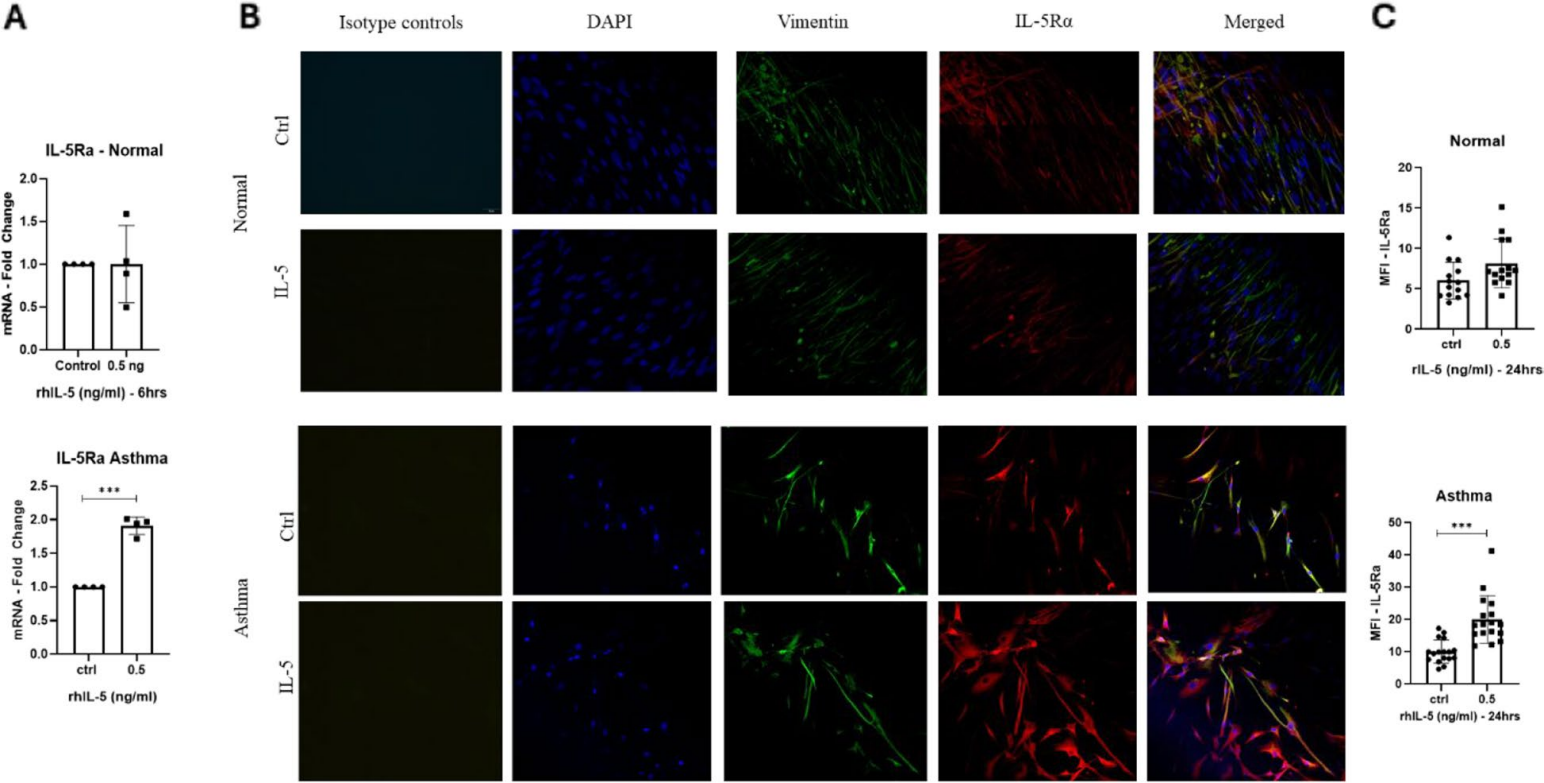
IL-5 inducing the expression of IL-5Ra in lung derived fibroblasts. **A** mRNA levels of *IL5RA* in both normal and asthmatic derived lung fibroblasts. *18s* was used as a housekeeping gene for ΔCt normalization, and fold changes upon IL-5 treatment compared to control was calculated as 2.^−ΔΔct^. Data shown are the mean ± SD of four separate experiment; two-tailed paired student’s t-Test statistical analysis was done * p < 0.05, ** p < 0.01, *** p < *0.001*. **B** Cultured normal and asthmatic fibroblasts, captured at 40X magnification, display immunofluorescence staining for IL-5Rα (green), vimentin (red), and DAPI (blue) in both cell types, along with mouse and rabbit IgG isotype controls. **C** Evaluation of the mean fluorescence intensity (MFI) of IL-5Rα expression in lung fibroblasts, quantified following 24 h of IL-5 stimulation. Statistical significance was determined using two-tailed paired Student's t-test based on data from three independent experiments, * p < 0.05, ** p < 0.01, and *** p < *0.001*

The literature highlights a contradictory role of IL-5 in the regulatory mechanism of IL-5Rα, [16–18]. To explore whether IL-5 exerts a similar effect on IL-5Rα expression in lung-derived fibroblasts, we assessed *IL5RA* mRNA levels after 6 h of stimulation. The qRT-PCR analysis revealed no significant effect on *IL5RA* expression in normal lung fibroblasts upon IL-5 stimulation. However, in asthmatic fibroblasts, *IL5RA* (1.91-fold, *p* = 0.0008) appeared to be significantly overexpressed in response to IL-5 stimulation (Fig. 5A). To validate these findings, an immunofluorescence assay was performed to evaluate IL-5Rα protein levels by measuring the mean fluorescence intensity (MFI) after 24 h of IL-5 stimulation. Consistent with the mRNA data, normal fibroblasts did not show an increase in IL-5Rα expression, whereas asthmatic fibroblasts exhibited a significant overexpression of IL-5Rα (*p* = 0.0005) (Fig. 5B-C). This receptor upregulation suggests a feedforward loop that potentiates IL-5 signaling, enhancing fibroblast activation in diseased airways.

### IL-5 induces distinct transcriptional profiles in lung derived fibroblasts

To delineate the transcriptomic mechanisms by which IL-5 drives pro-fibrotic reprogramming in asthma, we performed RNA sequencing on IL-5-stimulated lung fibroblasts from asthmatic and healthy donors, coupled with Gene Set Enrichment Analysis (GSEA), to identify dysregulated pathways underlying fibroblast activation and extracellular matrix remodelling (GSE285468). As positive control markers to validate the efficacy of IL-5 stimulation and the RNAseq results *FN-1* and *Col1A1* were used. The GSEA data confirmed an increase in expression of both following IL-5 stimulation (Supplementary Fig. 2).

Differential gene expression analysis revealed 472 genes differentially expressed between IL-5-stimulated asthmatic and healthy fibroblasts (Supplementary Fig. 3A-B, and Supplementary Table 2). Further, Gene Set Enrichment Analysis (GSEA) identified upregulated pathways, including MAPK signalling (NES = 1.8, *p* = 0.002) and ECM degradation (NES = 1.6, *p* = 0.01) (Supplementary Fig. 4B). These findings highlight IL-5’s role in reprogramming fibroblast function toward a pro-fibrotic phenotype.

**Fig. 6:**
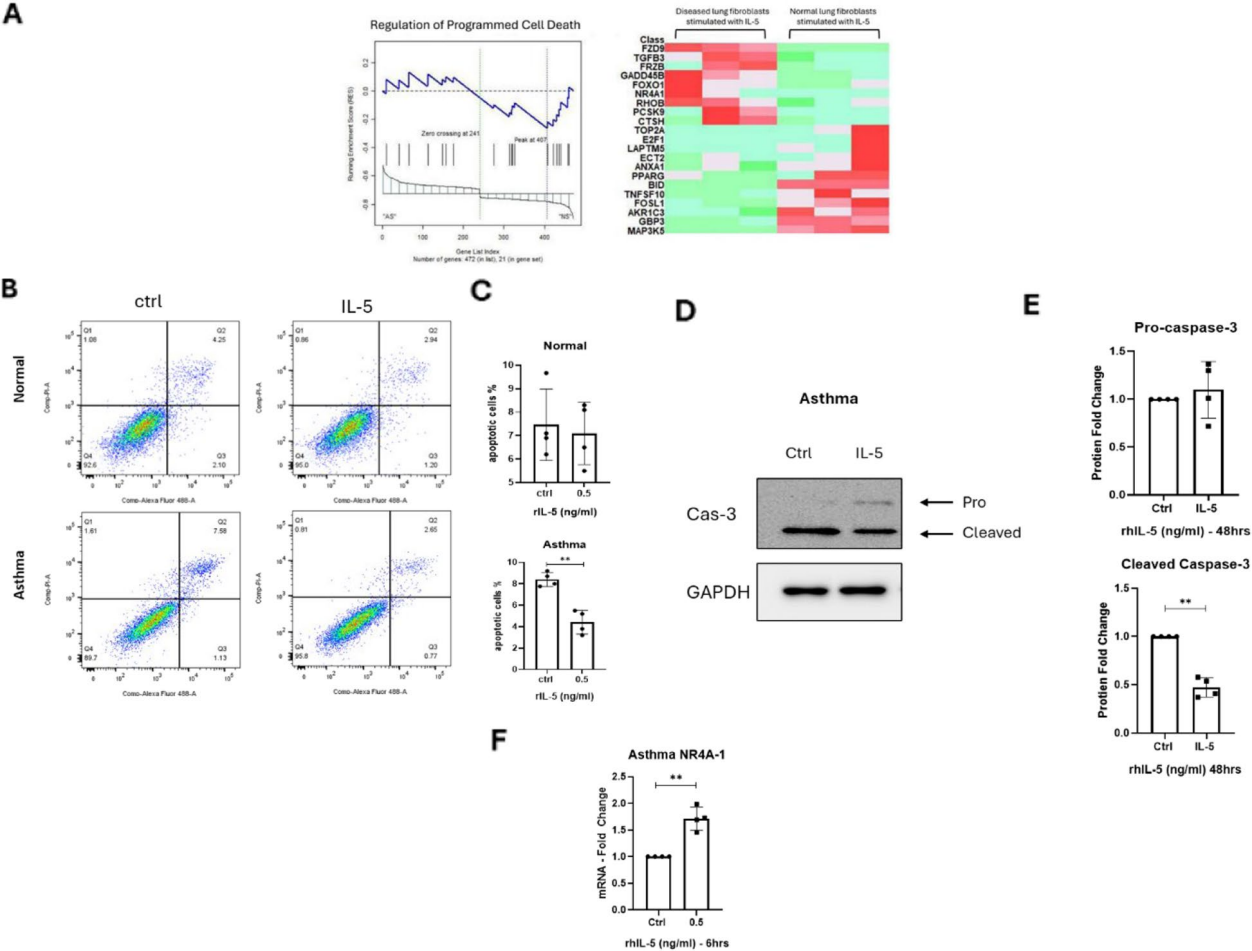
Lung derived *f*ibroblasts stimulated with IL-5 exhibit a *distinct transcriptional profiles* and enhances cellular survival*.* **A** Leading edge analysis showed a significant enrichment in the regulation of programmed cell death. The heatmap demonstrates the clustering of upregulated and down regulated genes, particularly *NR4A1*, *FZD9* and *FOXO1*; upregulated genes presented in red, down regulated genes presented in green. **B** Representative figure of Annexin V staining of both normal and asthma derived fibroblasts post 48 h of IL-5 stimulation, (**C**) bar graphs representing the percentage of both early and late apoptotic cells post IL-5 stimulation, data shown is the mean ± SD of four separate experiment, (**D**) representative immunoblots of Caspase-3 in asthma derived lung fibroblasts pre and post IL-5 stimulation, (**E**) Densitometric analysis of the immunoblots, proteins were normalized against GAPDH, data shown are the mean ± SD of four separate experiment; unpaired two-tailed student’s t-Test statistical analysis was done *p < 0.05, **p < 0.01*.* **F** The upregulation of the mRNA level of *NR4A1* post IL-5 stimulation in asthmatic derived fibroblasts, *18s* was used as a housekeeping gene for ΔCt normalization, and fold changes upon IL-5 treatment compared to control was calculated as 2.^−ΔΔct^. Data are the mean ± SD of four separate experiments; significance was determined using two-tailed paired student t-Test ** *p* < *0.01*

To investigate IL-5’s influence on fibroblast-microenvironment interactions, we employed absolute GSEA (absGSEA) to analyse its effects on pathways regulating extracellular matrix (ECM) dynamics and cellular sensitivity. Absolute GSEA (absGSEA) further implicated IL-5 in enhancing fibroblast responsiveness to environmental stimuli. Pathways associated with cellular migration (NES = 1.5, *p* = 0.01) and hormone regulation (NES = 1.4, *p* = 0.02) were enriched, while ECM organization pathways were paradoxically downregulated (Supplementary Fig. 4 A and C). These results suggest IL-5 primes fibroblasts for heightened interactions with their microenvironment, exacerbating tissue remodelling. Furthermore, the absGSEA analysis extends to encompass biological processes and immune responses, covering significantly identified pathways from curated gene sets (C2), computational gene sets (C4), Gene Ontology (C5), and cell type signature gene sets (C8). Our focus was oriented toward the C5 pathways representing significantly affected biological pathways, among activated pathways listed in Table 3, the primary focus was directed towards the programmed cell death pathway due to its relevance in the pathophysiology of tissue remodelling, particularly fibrosis (Fig. 6A).

**Table 3:** Gene ontology pathways obtained from absGSEA, GS = Gene Set, ES = Enrichment score, NES = normalized enrichment score, NOM *p*-val = nominal *P*-value

GS	SIZE	SOURCE	ES	NES	Gene %	NOM *p*-val	Tag %	Signal
Gene ontology: biological processes								
* Cell junction organization*	22	GO:0034330	0.36703	1.4228	0.146	< 0.001	0.318	0.285
* Positive regulation of catabolic process*	16	GO:0009896	0.37578	1.4225	0.288	< 0.001	0.438	0.322
* Regulation of programmed cell death*	21	GO:0043068	0.37859	1.2455	0.379	< 0.001	0.619	0.402
* Regulation of hormone levels*	21	GO:0010817	0.32879	1.1903	0.375	< 0.001	0.571	0.374
Gene ontology: molecular functions								
* Protein homodimerization activity*	22	GO:0042803	0.46241	1.695	0.322	< 0.001	0.591	0.42
* Protein dimerization activity*	31	GO:0046983	0.37892	1.4633	0.322	< 0.001	0.516	0.375
Gene ontology: cellular processes								
* Synapse*	30	GO:0045202	0.41766	1.5681	0.29	< 0.001	0.533	0.404
* Perinuclear region of cytoplasm*	26	GO:0048471	0.31735	1.3491	0.415	< 0.001	0.577	0.357

### IL-5 stimulation enhances cell survival through the inhibition of Programmed cell death

To further explore the differential regulation of programmed cell death in response to IL-5 stimulation, we performed absGSEA for enrichment analysis and obtained key genes involved in this pathway (Fig. 6A). The absGSEA results (left panel) indicated enrichment of the “regulation of programmed cell death” gene set in IL-5–stimulated normal lung fibroblasts compared to diseased fibroblasts. These findings suggest a stronger regulatory mechanism of apoptotic mechanisms in the normal cells when compared to their Asthmatic counter parts. Consistent with this, the heat map (right panel) illustrates clear expression differences across the 21 core genes that potentially contribute to this pathway. Genes such as GADD45B, FOXO1, TGFB3, FRZD9 and NR4A-1 showed higher expression in diseased fibroblasts, suggesting possible compensatory or altered regulatory responses. In contrast, the expression of BID, TNFSF10, FOSL1, and GBP3 were lower in these cells suggesting a dysregulation in the programmed cell death. For further confirmation, the full gene set that is related to the heat map is provided in the Supplementary material Table 3. For further confirmation, functional validation was performed using Annexin V staining assay, demonstrating the IL-5’s anti-apoptotic effects in asthmatic fibroblasts (*p* = 0.0057) (Fig. 6B–C), while Western blotting revealed a 0.6-fold decrease in cleaved caspase-3 (*p* = 0.0018), a hallmark of apoptotic suppression (Fig. 6D–E). To determine the mechanism behind the enhancement in the survival observed post IL-5 stimulation, the identified genes that were overexpressed from the heat map,NR4A1, a nuclear receptor strongly implicated in apoptosis inhibition, was significantly expressed in IL-5 stimulated asthmatic fibroblasts (Fig. 6A). qRT-PCR confirmed the significant upregulation of *NR4A1* in IL-5-treated asthmatic fibroblasts compared to controls (1.714-fold, *p* = 0.0072) (Fig. 6F). Altogether, our findings established that IL-5 sustains fibroblast survival by inhibiting apoptosis possibly through upregulating NR4A1 expression.

## Discussion

Asthma is a chronic inflammatory disease of the airways characterized by the interplay of multiple immune cells and their mediators [19]. One of the major pathological hallmarks of asthma is airway remodelling, which remains a significant clinical challenge to manage [3]. Airway remodelling encompasses structural changes in both small and large airways, including subepithelial fibrosis [20].

Our study provides novel insights into the fibrotic role of IL-5 in airway remodelling, focusing on its direct interaction with lung-derived fibroblasts. Traditionally, IL-5 has been well-characterized for its role in eosinophilic inflammation in asthma pathophysiology [9, 21]. However, our data demonstrate that both asthmatic and normal lung fibroblasts express functional IL-5Rα, with significantly higher levels in asthmatic fibroblasts. Furthermore, IL-5Rα signalling in fibroblasts is predominantly mediated through the RAS-ERK and PI3K-AKT pathways [11]. While IL-5Rα expression was previously thought to be exclusive to eosinophils and, to some extent, basophils [21], recent findings have reported its expression on airway epithelial cells [12] and lung fibroblasts [11]. The presence of IL-5Rα on fibroblasts suggests an additional role for IL-5 in airway remodelling, particularly in fibrosis [22]. IL-5 was found to enhance fibroblast proliferation, a key feature of fibrosis [4, 11].

We found that IL-5 stimulation significantly increased ECM mRNA and protein expression particularly of fibronectin, collagen I, although the mRNA levels of tenascin C was significantly elevated post IL-5 stimulation, but it didn’t match with the protein levels. This may be explained due to post-transcriptional regulation. Since fibroblasts are the primary cellular component of the subepithelial layer, an increased ECM turnover rate, characterized by excessive deposition and reduced degradation of ECM components such as collagen I, III, V, fibronectin, tenascin C, and lumican, contributes to fibrotic thickening and airway narrowing [8]. This pathological ECM remodelling is known to exacerbate airway obstruction in asthma [23].

Another key contributor to fibrosis is the imbalance between MMPs and their tissue inhibitors TIMPs. Our study highlighted the significant upregulation of MMP-3 and MMP-2 following IL-5 stimulation. Pathologically, disruptions in the 1:1 ratio between MMP and TIMP led to aberrant tissue repair [24]. Despite the outcome of the enrichment data that reveals a high ECM degradation rate, our in vitro data is showing an elevation in the levels of MMP-2, MMP-3 as well as TIMP-1 and TIMP-2 that results in an MMP/TIMP enrichment. Reports are showing contradictory roles of MMP-2 in the context of asthma, Takahashi et al. is showing that it has a protective role in terms of airway hyperresponsiveness, while Kuwabara and his colleagues showed that higher levels of MMP-2 is associated with elevated levels of myofibroblast migration to the airway thus enhancing fibrosis [25]. Although MMP-9 has been extensively linked to asthma severity, as its levels in sputum and lung tissue correlate with disease severity [26, 27], our study did not observe changes in MMP-9 following IL-5 stimulation. However, increased TIMP-1 levels suggest an overall MMP/TIMP enrichment that favours ECM accumulation and fibrosis. Notably, MMP-3 has been linked to collagen I levels and negatively correlated with Forced Expiratory Volume in one second (FEV1) [28, 29]. Additionally, MMP-3 promotes airway smooth muscle (ASM) cell migration [23], further exacerbating remodelling.

IL-5 stimulation also enhanced the secretion of IL-6, a key immune cytokine, and TGF-β, a potent pro-fibrotic growth factor. Fibroblasts, while primarily structural cells maintaining the subepithelial layer, contribute to airway inflammation by secreting cytokines such as IL-6, TNF-α, TGF-β, and IL-1β [30]. Although IL-6 is well known for its role in polarizing CD4-T cells toward Th17 phenotype [31], however other studies showed that IL-6 is involved in skewing naive T cells toward a Th2 phenotype, further promoting IL-4 secretion and perpetuating the type 2 immune response [32], as well as inducing the secretion of IL-13 but not IL-5 [33], on the other hand, IL-6 can also act on non-immune cells by directly stimulates lung fibroblasts enhancing collagen deposition as well as TIMP-1 expression, further favouring fibrosis [34, 35] This prolonged inflammatory state sustains remodelling and abnormal tissue repair. TGF-β is well-established in asthma pathophysiology for its role in fibroblast activation, myofibroblast differentiation, and ECM deposition [36], the induction of TGF-β in response to IL-5 might also contribute alongside with IL-5 in the ECM expression Notably, IL-5-induced IL-6 [37] and TGF-β [38] secretion could contribute to steroid resistance in severe asthma, explaining why airway remodelling — driven in part by fibroblasts that respond poorly to corticosteroids [39] — remains difficult to manage with conventional therapy [37, 38].

The regulatory effects of IL-5 on its receptor remain controversial. While IL-5Rα is upregulated in eosinophils upon persistent allergen exposure [40], studies have also suggested a downregulatory effect of IL-5Rα due to MMP activity [16]. Other reports indicate that IL-5Rα regulation depends on eosinophil maturation and localization [18]. Our study found that IL-5 upregulated its receptor specifically in asthmatic fibroblasts, potentially sustaining a positive feedback loop that deviates from classical eosinophilic regulatory mechanisms.

Pathway enrichment analysis revealed significant associations with cytokine signalling and immune response pathways, including "cellular response to cytokine stimulus" and "cytokine signalling in the immune system." These findings reinforce the critical role of immune mediators in asthma pathophysiology [41, 42]. Our transcriptomic analysis further suggests that IL-5 amplifies fibroblast responsiveness to surrounding cytokines, particularly in T2-high asthma, where IL-5 levels are elevated [43]. Additionally, IL-5 significantly enhanced asthmatic fibroblast migration, mirroring the effects of other pro-fibrotic cytokines and proteins such as TGF-β and periostin, respectively [44, 45]. This suggests that IL-5 may synergize with other cytokines such as IL-4 and IL-13 that are known to have a fibrotic by stimulating airway fibroblasts, further exacerbating airway remodelling [46], positioning it as a potential therapeutic target for fibrosis-related complications in asthma.

Interestingly, our gene ontology analysis identified an upregulation of genes associated with fibroblast survival, particularly *NR4A1* which was accompanied with downregulation of *BID* a pro-apoptotic gene [47]. IL-5 stimulation inhibited apoptosis in asthmatic fibroblasts, with a notable downregulation of cleaved caspase-3. While NR4A1 exhibits contradictory roles in apoptosis [48, 49], our findings suggest its anti-apoptotic function in asthmatic fibroblasts [50]. These results highlight IL-5's dual role as an activator of fibroblasts, promoting both ECM deposition and survival.

Targeting IL-5 with biologics such as mepolizumab has shown efficacy in reducing eosinophilic inflammation [51] as well as reducing the sub-basement membrane thickness and the airway smooth muscle area [52] However, our findings suggest that anti-IL-5/IL-5Rα therapies may also mitigate fibroblast-driven fibrosis, addressing a critical unmet need in severe asthma management.

The limitation of this study is the relatively small sample size. Although the isolated cells were derived from obese asthmatic patients, which may limit the generalizability of the results to all asthmatics, this nonetheless highlights the relevance of our findings, as obesity is commonly linked with a more severe asthma phenotype [53, 54]. In addition to the use of rigorously phenotyped asthmatic fibroblasts strengthens the internal validity of our results. Future studies with larger cohorts and in vivo models are needed to confirm IL-5’s direct role in airway remodelling. Additionally, investigating the regulation of IL-5Rα in fibroblasts and its crosstalk with other cytokines, such as TGF-β, could uncover synergistic pathways driving fibrosis.

## Conclusion

Our study provides novel insights into the role of IL-5 in airway remodeling by demonstrating its direct effect on lung-derived fibroblasts. We show that IL-5 stimulation enhances extracellular matrix deposition, upregulates fibrotic mediators including collagen I, Fibronectin and Tenascin C, and promotes fibroblast cells survival, contributing to the persistent structural changes observed in asthma. These findings suggest that IL-5 signaling extends beyond its well-established role in eosinophilic inflammation and may play a direct role in fibrosis-associated airway remodeling.

Given the clinical significance of airway remodeling in asthma, targeting IL-5 or its downstream signaling pathways in fibroblasts may represent a potential therapeutic strategy. Future studies employing in-vivo models and patient-derived samples are necessary to further validate these findings and explore the translational potential of IL-5 blockade in mitigating fibrotic airway changes.

## Supplementary Information

Supplementary Material 1

Supplementary Material 2

## Data Availability

The RNA sequencing data generated in this work have been deposited in the Gene Expression Omnibus (https://www.ncbi.nlm.nih.gov/geo (accessed on 26. December 2024) under GEO Series access number GSE285468 and can be accessed from https://www.ncbi.nlm.nih.gov/geo/query/acc.cgi?acc = GSE285468 (accessed on 11 April 2025). All other supporting data of this study are either included in the manuscript or available on request from the corresponding author.
