# Supplementary material for: IL-5 signaling in asthmatic derived fibroblasts exacerbates airway remodeling through ECM dysregulation and apoptosis resistance
Source: Respir Res. 2025 Nov 4;26:307. doi: 10.1186/s12931-025-03371-x (PMC12584347; doi:10.1186/s12931-025-03371-x)
Supplement: Supplementary file 1 — Supplementary Material 1 [file 12931_2025_3371_MOESM1_ESM.pdf]

Supplementary Figure 1.

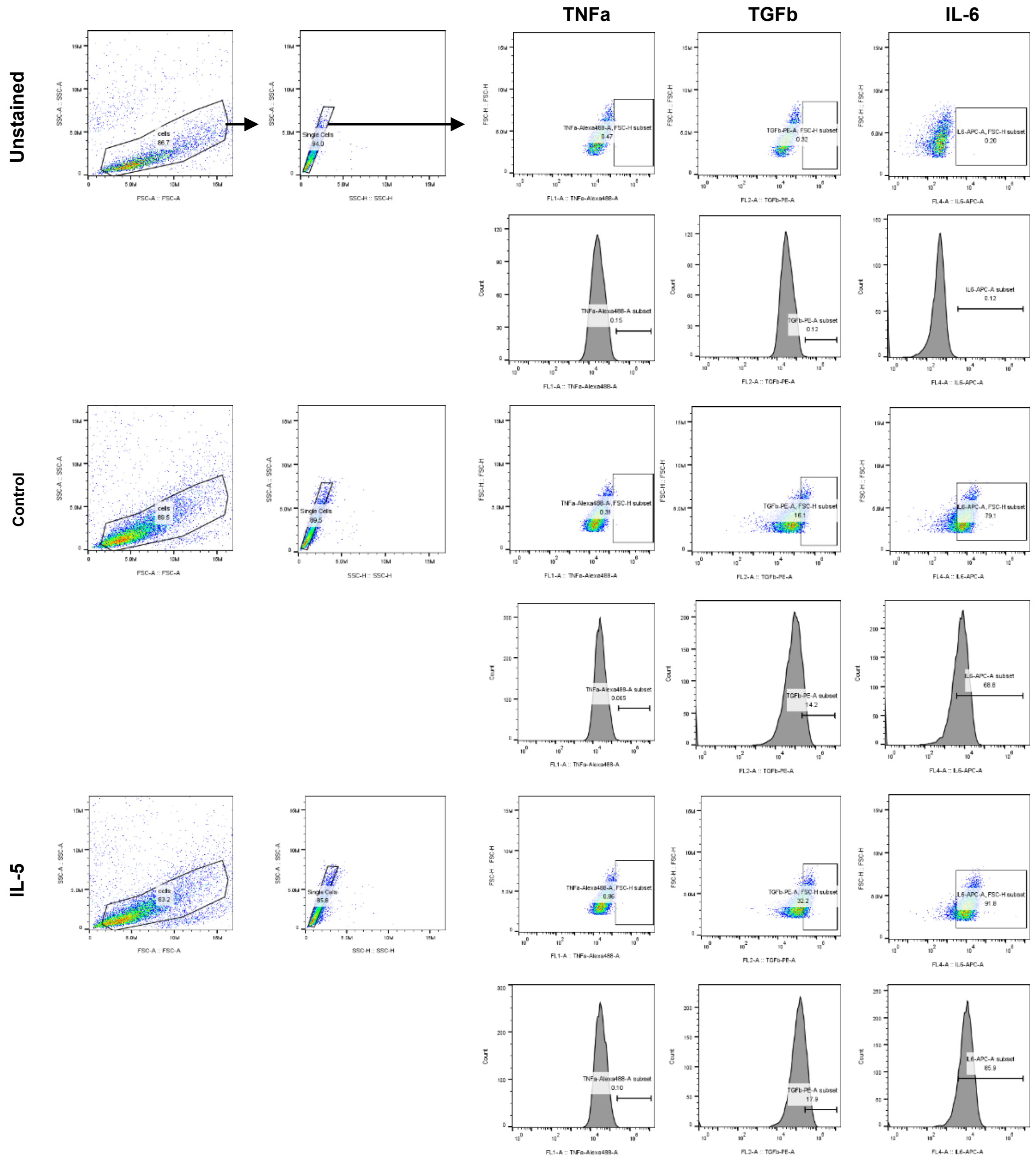

Supplementary Figure 1: Representative gating strategy for intracellular cytokine staining of lung fibroblasts following IL-5 stimulation.

Cells were first gated based on forward scatter (FSC-A) and side scatter (SSC-A) to exclude debris. Singlets were identified by FSC-A, and live cells were selected using viability dye exclusion. Cytokine-positive populations were then gated based on fluorescence intensity. This strategy was applied based on the control and copied to the other samples; each experiment had its own controls.

Supplementary Figure 2

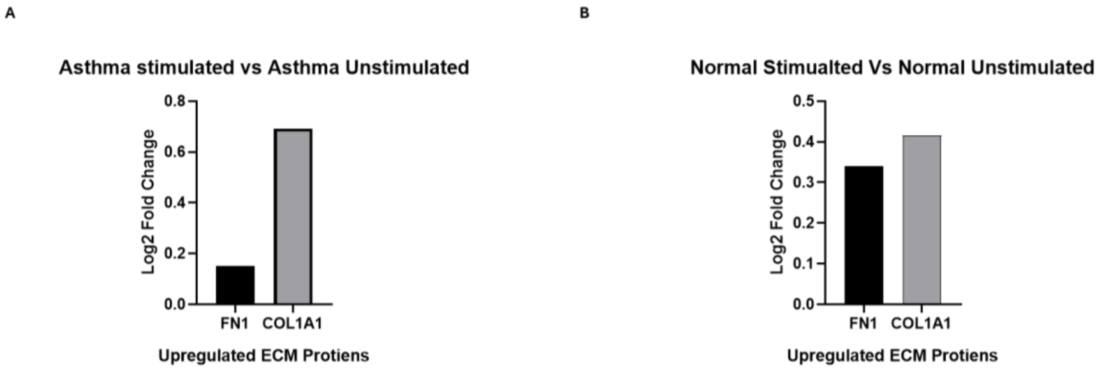

Supplementary figure 2: FN1 and Col1A1 as validation controls.

(A)The expression of FN1 and Col1A1 in asthma stimulated with IL-5 based on Log2Fold change. (B) The expression of FN1 and Col1A1 in Normal stimulated with IL-5 based on Log2Fold change.

Supplementary Figure 3

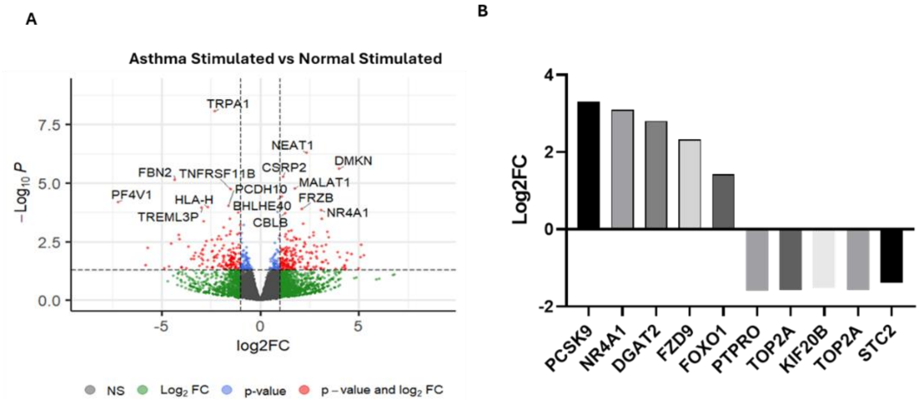

Supplementary figure 3: Lung derived Fibroblasts stimulated with IL-5 exhibit a Distinct Transcriptional Profiles.

(A) Volcano plots of differentially expressed genes between asthmatic and normal derived fibroblasts stimulated with IL-5.
(B) Genes that are differentially expressed in response to IL-5 stimulation based on the obtained Log2FC.

Supplementary Figure 4

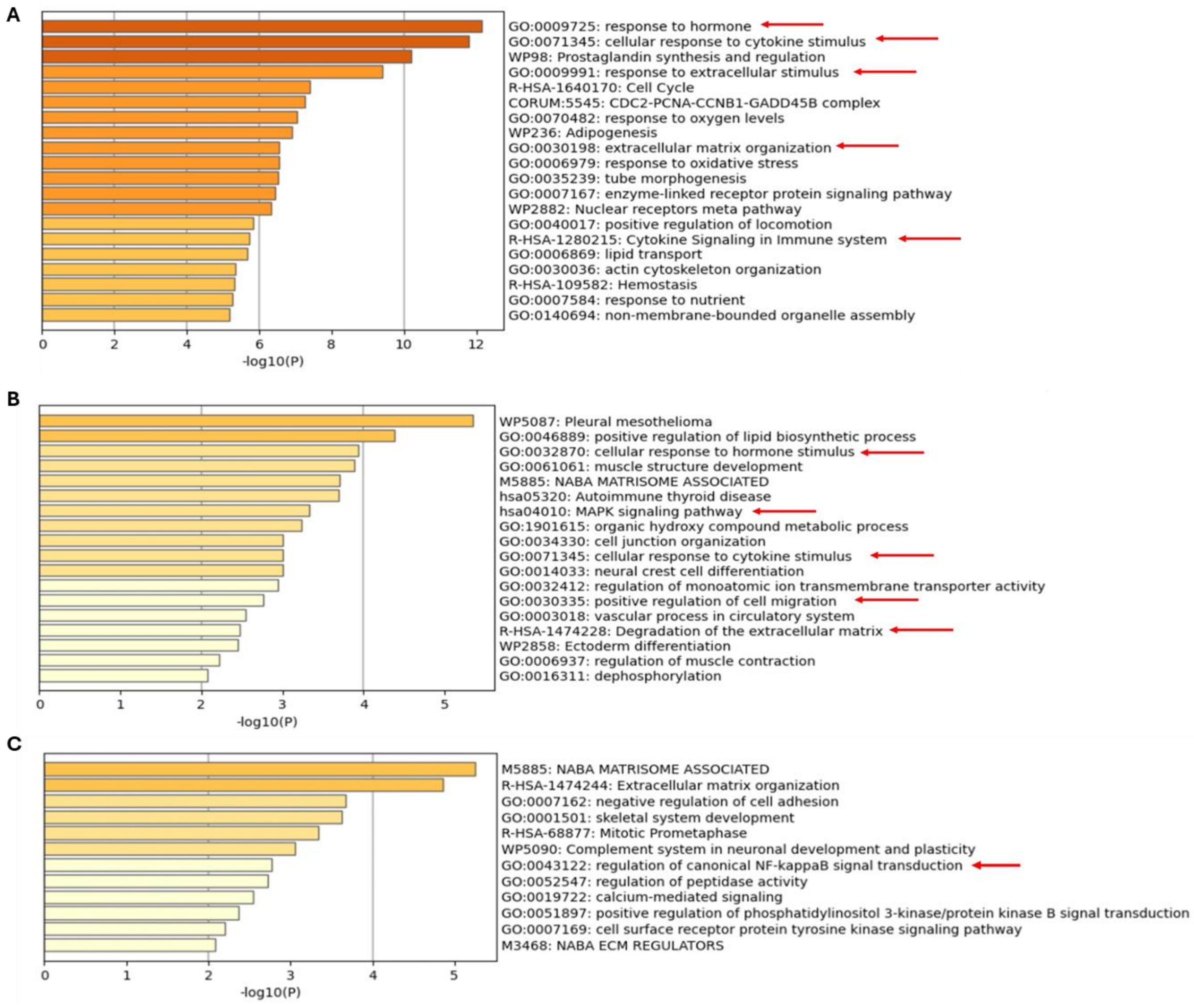

Supplementary figure 4: Gene set enrichment analysis of fibroblasts post stimulation with IL-5.

(**A**) Enriched pathways in asthmatic derived fibroblasts post IL-5 stimulation **(B)** Upregulated pathways in asthma Stimulated based on log2FC > 2 **(C)** Downregulated Pathways in asthma Stimulated based log2FC <2.
